# Caring soils for sustainable land uses

**DOI:** 10.1111/1751-7915.13631

**Published:** 2020-07-21

**Authors:** Juan‐Luis Ramos, Rocio Lansac

**Affiliations:** ^1^ Estación Experimental del Zaidin–CSIC Granada Spain; ^2^ Instituto Nacional de Investigación y Tecnología Agraria y Alimentaria (INIA) Madrid Spain

## Abstract

The aim of the article is to develop and explore the idea that soil health is an essential element in combating climate change and promoting food security. An important aspect of this is that soil, rather than simply serving to support plants and as a niche for animals and microbes, also functions as a natural reactor that, through a series of chemical and biological reactions, purifies water, replenish aquifers and maintains equilibria in surface waters. This topic is particularly timely given the recent announcement of the Mission program for Health of Soils by the European Commission. Within this realm, the article intends to catalyze and promote the debate around what defines sustainable agriculture in order to help shape its future.

Soil is a non‐renewable resource that enables the production of food, feed, fibre and wood. It also serves as a key player in the purification of ground water, and is a habitat in which microbes thrive and carry out the biogenic cycles needed for life. Our society is becoming aware of the devastating effects of global warming and the need for reducing pollution (i.e. the recent focus on reducing plastic waste in the ocean). However, awareness about the need for building and maintaining healthy soils is not as widespread. This lack of awareness is worrying given that it has been estimated it takes nearly a century to add just three millimetres of new topsoil. This, combined with the increasing demand for food production as the population grows (i.e. the human population is projected to reach 10 billion by 2050), makes a strong case for the need for long‐term strategies to preserve and build healthy topsoil.

Soil is a dynamic system that exhibits a complex interplay and equilibrium between physicochemical and biological characteristics. A dizzying array of chemical reactions takes place in soil – so many so that it likely represents the largest chemical reactor on our planet. Healthy soil is vital for the purification of waters and the recharging of aquifers. While it is widely known that plants fix CO_2_, it is less widely known that soil is a key CO_2_ sink; around 20% of the carbon fixed by plants is released as exudates into the surrounding soil, where it enriches the organic matter and enhances soil quality. The United Nations and the European Commission have emphasized the importance of proper soil management, while discouraging agricultural practices that promote greenhouse gas emissions. Biodiversity in our planet refers not only to the plants and animals that we can see with our eyes, but also to the immense reservoir of microscopic life present in soils. In fact, a single gram of the soil adjacent to the roots of plants – known as rhizosphere soil – can contain anywhere between 10 and 100 million microbes, while bulk soil can contain between 10 000 and 1 million microbes per gram. The diversity of these microbes is so immense that many authors claim that <1% of the all soil microbes have been cultivated in the laboratory. In fact, current metagenomic approaches are only now revealing the degree of this rich microbial biodiversity, as well as how different microbes function together as a larger community via coupled chemical interactions. This emerging area of research is advancing the limits of our understanding of the complexities of biogenic cycles and the interactions (e.g. microbe–microbe, microbe–plant and microbe–insect interactions) that make these cycles possible.

Soil problems may be seen as local issues, but the United Nations has already indicated that 33% of land not covered by snow is affected by desertification; furthermore, it is estimated that around 25% of agricultural soils in Europe are severely compromised. Considering climate change and the breath of its effects, it is clear that local actions have a global impact and that policies need to take a global view to ensure the health of our planet – as we have all recently seen during COVID pandemic. In this line it is worth considering that UN Sustainable Development Goals seek "to protect, restore and promote sustainable use of terrestrial ecosytems, sustainably manage forest, combat desertification and halt and reverse biodiversity loss".

Ensuring the health of soils is a key for safeguarding food production – especially considering that healthy soils are less prone to attacks from plant pathogens. Plant diseases and plagues currently cost us around 20%–30% of our crops. Insufficient food production creates local famine as well as serving as a catalyst for dramatic and uncontrolled human migrations. Sustainable soil practices are also important for the creation of rural jobs, which are an important part of attaining balanced rural‐urban growth.

During the Climate Change summit held in December 2019 in Madrid, the European Commission announced new support for R&D programs focused on soil health. The Commission flagged co‐design as an important part of the program. Co‐design enables collaboration between diverse local, national and international partners and is a key for improving soil health in the short (2030) and mid‐terms (year 2050). By fully engaging all stakeholders, including consumers, farmers, NGOs, scientists, stakeholders and politicians, co‐design creates fertile ground for ideas. The approach relies on what are called lighthouses farms – these sites serve as living laboratories to test and validate new approaches gained from the co‐design process. They comprise of smaller field plots from which successful approaches can be launched into larger testing grounds. It is important to note that that projects must also consider how these approaches are integrated within the greater landscape, while considering recreational and cultural implications – factors that affect adoption of the strategies and ensure lasting impacts.

The current COVID crisis has revealed the vulnerability of our society and the need to carefully consider the long‐term effects of our actions on the natural systems upon which we depend. It is only through this inclusive and broad lens that we will ensure the sustained prosperity of generations to come.

## Conflict of Interest

None declared.

